# Spinal Anesthesia and Spinal Anesthesia with Subdiaphragmatic Lidocaine in Shoulder Pain Reduction for Gynecological Laparoscopic Surgery: A Randomized Clinical Trial

**DOI:** 10.1155/2017/1721460

**Published:** 2017-08-08

**Authors:** Zahra Asgari, Mahroo Rezaeinejad, Reihaneh Hosseini, Masoumeh Nataj, Maryam Razavi, Mahdi Sepidarkish

**Affiliations:** ^1^Department of Obstetrics and Gynecology, Arash Women's Hospital, Tehran University of Medical Sciences, Tehran, Iran; ^2^Department of Epidemiology and Reproductive Health, Reproductive Epidemiology Research Center, Royan Institute for Reproductive Biomedicine, ACECR, Tehran, Iran

## Abstract

**Objective:**

The aim of this study was to compare the effectiveness of spinal anesthesia with subdiaphragmatic lidocaine at the beginning of surgery versus spinal anesthesia in pain reduction for gynecological laparoscopic surgery.

**Methods:**

This was a clinical trial conducted in Arash Hospital, Tehran, Iran. Eighty-four patients were randomized to either spinal anesthesia with subdiaphragmatic lidocaine, spinal anesthesia, or general anesthesia (GA). The primary outcome was patients' pain perception during surgery, 2, 4, 6, and 12 hours after surgery, and prior to discharge and was assessed by visual analogue scale (VAS).

**Results:**

The results showed that there are no significant changes in pain perception over time in none of the three groups (*F*(4,76) = 0.37, *P* = 0.82). The severity of pain experienced by patients at all-time interval after surgery was similar between groups [*F*(2,79) = 0.54, *P* = 0.58].

**Conclusion:**

The use of subdiaphragmatic lidocaine at the beginning of surgery combined with spinal anesthesia was not associated with a statistically significant difference in patients' postoperative VAS scores compared to spinal anesthesia and GA during and after gynecological surgical procedures. The study was registered in Iranian Registry of Clinical Trial by the number of IRCT2016022226698N1.

## 1. Introduction

Laparoscopic surgery for women traditionally is done under general anesthesia, but nowadays spinal anesthesia is appropriate alternative with few complications in the surgery [1]. Spinal anesthesia is associated with numerous benefits, including ease of doing the procedure, low probability of failure, patient's consciousness, feeling less pain after surgery, lower intake of analgesics after surgery, early discharge, and avoidance of possible risks of general anesthesia and intubation [2]. However, spinal anesthesia is accompanied with neurologic deficits, such as cauda equina syndrome, low blood pressure, bradycardia, headache, and back and shoulder pain [2, 3]. The complications will be less observed if an appropriate dose is selected, anesthesia level is controlled, and sterilization is met [4]. The most important problem of laparoscopy under spinal anesthesia is Trendelenburg's position and upper abdominal pressure, which lead to neck and shoulder pain in a few minutes after starting the pneumoperitoneum, caused by nervous stimulation of the diaphragm and phrenic nerve [5]. The problem may result in stress in patients [6]. It is likely that the pain would be uncontrollable and consequently the surgery is impossible [2]. According to the pathophysiology of the problem, one of the suggested solutions is a local anesthetic infiltration in the subdiaphragmatic area at the beginning of surgery [7]. To the best of our knowledge, the technique has not been considered. The aim of this study was to compare the effectiveness of spinal anesthesia with subdiaphragmatic lidocaine at the beginning of surgery versus spinal anesthesia and general anesthesia in pain reduction for gynecological laparoscopic surgery.

## 2. Material and Methods

This study was designed as a single-center, randomized, parallel-group, controlled trial in accordance with Consolidated Standards of Reporting Trials (CONSORT) guidelines. The study was approved by Institutional Review Board of the Tehran University of Medical Sciences. Women attending the Department of Obstetrics and Gynecology, Tehran University of Medical Sciences, at Ruin Tan Arash Hospital for infertility treatment were recruited when the following criteria were met: (a) American Society of Anesthesiologists (ASA) physical status I-II, (b) age of 15–45 years, and (c) a signed informed consent form. Exclusion criteria included (a) severe coagulopathies, (b) severe cardiovascular diseases, (c) allergy to local anesthetics, (d) pathologies connected with abdominopelvic pain that could confuse the perception of pain directly related to the procedure (e.g., endometriosis), (e) chronic pain syndromes, (f) BMI > 35, (g) a history of abdominal surgery, (h) a history of psychological diseases, (i) being unable to comprehend visual analog scale, and (j) patients in disagreement with the study protocol. The written informed consent was obtained from the patients before conducting any study-related tests. The study was registered in Iranian Registry of clinical trial (http://www.IRCT.ir) by the number of IRCT2016022226698N1.

Women were randomized into three groups by means of a random table generated by computer-generated random numbers based on a block size of 6. The randomization list was kept in sealed, sequential opaque envelopes, which were opened after admission, and the treatment was administered before the patient enters the operating room.

A project nurse who was not involved in the operation evaluated each patient's pain and the presence of postoperative side effects.

Pain levels were also assessed throughout using a 100 mm linear VAS (0 = none, 100 = most painful). The pain VAS is a continuous measurement instrument that is operationally comprised of a horizontal line, anchored at each end by verbal descriptors, such as “no pain” and “the worst pain imaginable.” Visual analog scale (VAS) is a robust and reliable instrument which has been regularly used in similar trials by multiple investigators for different procedures.

### 2.1. Intervention

Following our standard practice, all patients were premedicated with oral midazolam 0.05 mg/kg. Standard monitoring, including electrocardiography, noninvasive blood-pressure measurements, and pulsoximetry, was undergone throughout the operation.

#### 2.1.1. Interventions in Groups A (Spinal Anesthesia) and B (Spinal Anesthesia with Subdiaphragmatic Lidocaine)

In the operating room, an IV cannula was inserted; then a 10 mL kg^−1^ Ringer's lactate solution was administered before the spinal block commences. Afterwards, the subarachnoid space was entered from the L3-4 or L4-5 interspace using a 25-G Quincke spinal needle in the sitting position. Once the cerebral spinal fluid (CSF) flow had been observed, 4 mL of hyperbaric bupivacaine 0.5% was injected into the subarachnoid space at a rate of 0.1 ml/s. The patient was then positioned supine, with a right hip pad to minimize aortocaval compression. The level of sensory block was tested using the gentle pinprick method and recorded.

Patients in Group B received subdiaphragmatic injections of 10 mL of 1% lidocaine at the port sites at the beginning of the procedure.

#### 2.1.2. Intervention in Group C (General Anesthesia)

For the induction of anesthesia, we used midazolam (0.02 mg/kg) (Chemidarou Industrial Company Tehran, Iran), propofol (1% 1–2.5 mg/kg) Liporo 1% (B. Broun AG Co., Germany), and fentanyl (1-2 *μ*g/kg) (Aburaihan Co. Iran) and for endotracheal intubation we used succinylcholine (1.5 mg/kg b.w.). For maintenance of anesthesia, propofol 100–150 *μ*g/kg (B. Broun AG Co., Germany) was administered. During the operation, atracurium 10 mg and fentanyl 50 *μ* were given every 30 minutes.

Laparoscopic procedures were performed using the standard two-puncture technique with carbon dioxide and Filshie clips. A subumbilical skin incision was made and a 3 mm trocar was pushed directly into the peritoneum. Then, a 2.9 mm mini endoscope (Karl Storz, Germany) with a 0-degree view was inserted, insufflating approximately 2 L of CO_2_. One lateral 3 mm port was also inserted using a mini grasper, fallopian tubes, and other pelvic organs exposed. Chromopertubation was performed by injecting 30 mL of methylene blue through an intrauterine Foleys catheter.

### 2.2. Sample Size and Statistical Analysis

The calculation of the sample size was based on the primary end point (VAS score). The study was designed to have 80% power to detect a 35% difference in pain scores on visual analog scale, with two-sided alpha levels of 0.05. Using sample size calculation for independent proportions, the minimum number of participants in each group should be 28 (totally 78 participants).

### 2.3. Data Analysis

All data analyses were conducted using SPSS version 21.0 for Windows (SPSS Inc., Chicago, IL, USA). Descriptive statistics for continuous variables were presented as mean ± standard deviation (SD) and for categorical variables as numbers (percentage). The baseline characteristics of the three groups were compared using analysis of variance test (ANOVA) for continuous variables and the Chi-square test for categorical variables. The endpoint mean VAS score was analyzed using repeated measures analysis of variance. The model included treatment as fixed factor and age and parity as covariates. All statistical tests were two-sided and the level of statistical significance was set at 0.05. All analyses were performed on an intent-to-treat basis. The conduct and analysis of the trial adhered to the 2010 CONSORT guidelines.

## 3. Results

**Figure 1:**
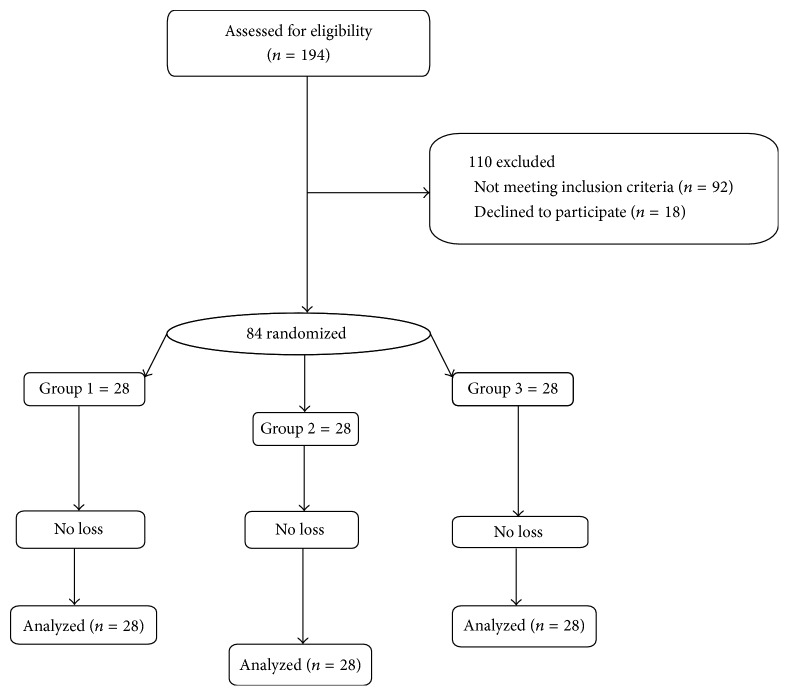
Flowchart showing participants' recruitment. Group 1: general anesthesia, Group 2: spinal anesthesia, and Group 3: spinal anesthesia with lidocaine.

Between April 2016 and July 2016, 194 patients were enrolled in the study. Of the 84 eligible patients, 28 patients were allocated to each of the treatment groups. The analysis was done by intention-to-treat and included all patients who were randomly assigned.  Figure 1 shows the trial profile. The three groups were matched, with no significant differences regarding the mean age, parity, weight, height, and indication for laparoscopy. Baseline demographic and clinical characteristics are summarized in Table 1.

**Figure 2:**
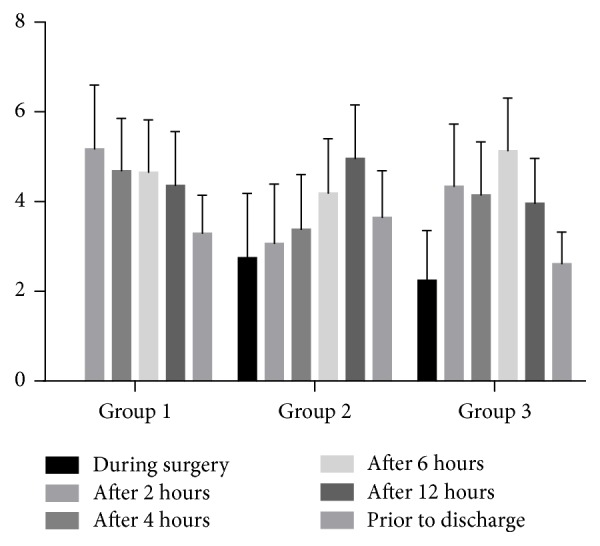
The results of reported pain experienced at all-time interval. Group 1 = general anesthesia, Group 2 = spinal anesthesia, and Group 3 = spinal anesthesia with lidocaine.

The severity of pain experienced by patients at all-time interval after surgery was presented in Table 2 and Figure 2. Although the mean of pain was different between groups, this difference was not statistically significant [*F*(2,79) = 0.54, *P* = 0.58].

Although the mean of pain increased for all groups during the study, repeated measures ANOVA revealed that there are no significant within-subject effects, indicating no significant change over time in none of the three groups (*F*(4,76) = 0.37, *P* = 0.82).

Compared with spinal anesthesia group, pain score during surgery was similar in spinal anesthesia with subdiaphragmatic lidocaine (Figure 2).

There were no statistically significant differences between the three groups with regard to narcotic analgesic usage (*P* = 0.84) and vomiting (*P* = 0.94).

## 4. Discussion

The objective of the present study was to investigate the effect of subdiaphragmatic infiltration of lidocaine 1% at the beginning of surgery combined with spinal anesthesia in order to reduce pain after less invasive surgery in women. In contrast to results of previous studies, findings of the present study showed that mean severity of pain in the patients in three groups studied immediately after the surgery, and 2, 4, 6 and 12 hours after surgery, and at the time of discharge was not statistically significant. Also, adding lidocaine 1% at a rate of 10 cc to the area below the diaphragm at the beginning of surgery did not affect severity of shoulder pain, which was similar to those who were under spinal anesthesia alone. Intraperitoneal instillation of local anesthetic was effective in controlling pain after appendectomy and cholecystectomy in the previous studies [5, 8, 9]. In a meta-analysis study conducted on 24 randomized clinical trials (RCTs), it was found that local anesthetic into the peritoneal area is statistically effective and safe in reduction of laparoscopic cholecystectomy [8]. However, instillation of local anesthetic into the peritoneal area is considered pain reduction after laparoscopic surgery. Some researchers confirmed the procedure for pain reduction, but others did not [10–15]. In a systematic review by Marks et al. in 2012, seven studies finally were included in meta-analysis. Inclusion criteria were use of local anesthetic compared with a placebo group. In general, results showed that infiltration of local anesthetic into intraperitoneal area is effective only at the first 6 hours after surgery, but there was no significant difference in patients' pain between intervention and placebo groups 24 hours after surgery. Each of 7 studies compared pain at one and two hours after surgery between intervention and placebo groups. Totally, 220 patients and 171 controls were included in intervention and placebo groups, respectively. The treatment group reported less pain than the placebo group (weighted mean difference, −1.82; 95% CI: −2.55 to −1.08). Three of seven studies examined the impact of the intervention on pain at 4 to 6 hours after surgery. In the treatment group, 108 patients received the intervention and 57 subjects were included in the placebo group. The intervention group reported less pain than the placebo group (weighted mean difference, −2.00; 95% CI: −3.64 to −0.35). Three other studies also compared pain at 24 hours after surgery between the two groups. In these studies, 106 subjects received treatment and 47 subjects were in the placebo group. There was no significant difference between the two groups (weighted mean difference, −0.26; 95% CI: −0.88 to 0.35) [16]. In the current study, the effect of local anesthetic on pain reduction was not observed because of having no placebo group (without anesthesia). In our study, the effectiveness of local anesthetic added to spinal anesthesia compared with spinal and general anesthesia. Therefore, it is expected to not have any relationship caused by dilution of difference between groups.

Spinal-epidural anesthesia is commonly used in surgery for women, and consequently neck and shoulder pain after surgery using the anesthesia is the most important concern [17]. Previous studies revealed that the factors influencing the pain are multifactorial, such as local trauma by epidural needle, high body mass index (BMI), patient's position during surgery, duration of surgery, and epidural instillation times [18]. Pain after laparoscopic surgery is divided by three parts including incision pain, resulting in visceral pain and neck and shoulder pain [14]. The rationale for intraperitoneal anesthesia is that it blocks the free afferent nerve endings in the peritoneum; however, absorption from the peritoneal surface also acts as an additional mechanism of analgesia. When local anesthetics are applied directly to the site of injury on the peritoneal surface, there is faster resumption of bowel motility. This is due to reduced neuroendocrine response to surgery; in addition, the local anesthetics act directly on smooth muscle cells of the bowel wall [19].

In research on effectiveness of local anesthetics on pain reduction, there was no agreement based on the type, dose, and local anesthetic infiltration. In several studies, infiltration of trajectory of the trocars, infiltration of fallopian tubes, and peritoneal instillation before and after insufflations have been suggested by gas [20].

In the current study, duration of surgery in three groups of intervention was approximately 30 minutes and three groups had no statistically significant difference together. Although the exact length of efficacy of topical lidocaine is unknown, most likely it is more than a half-life of 2 hours of injected type. Considering a strong relationship between the dose of anesthetics and anesthesia intensity, one of the reasons that we were not able to see the effects of local anesthetic was low dose of the anesthetic [12]. In most studies that evaluated positive effects of local anesthetic, the dose was high. Goldstein et al. reported that 20 ml infiltration of bupivacaine 5% or ropivacaine 75% reduced pain and consumption of analgesic compared with placebo [21]. In another study, Callesen et al. found that infiltration of ropivacaine (50 ml) in the area between the peritoneal and mesosalpinx may have a positive role in reducing pain in 80 patients who underwent laparoscopic tubal sterilization to eliminate infection [22].

The results of our study were different compared with findings of previous studies. One of the differences was a different pain assessment tool. Although in previous studies VAS was the most used tool, tools like Wong-Baker Faces Pain Rating Scale (WBFS) and Modified McGill Pain intensity scores also were used in other studies [23–26]. With regard to the outcome of pain as a subjective construct, one can assume that there is measurement error in various studies and could be one of the reasons for difference between the results of current studies and other studies [19].

Given that the time of these studies is different and from 1980 until now no search has been conducted, the effect of the development of surgical techniques and anesthesia may be the reason for heterogeneity of results of studies [16].

Although the majority of studies that were retrieved in the literature indicated the effectiveness of local anesthesia on pain after surgery, in three RCTs, similar to our study, there was no effect of local anesthesia on pain. All of three RCTs were conducted after a systematic review by Marks et al. in 2012. In a study conducted in 2016 by Collins et al., 55 patients who underwent laparoscopic and robotic gynecologic procedures were women and were assigned to two groups of intraperitoneal ropivacaine and placebo. Pain intensity was measured 2, 4, 8, and 12 hours after surgery. However, the mean pain score in the placebo group was more than the treatment group, but this difference was not statistical significant [27]. In a study by Andrews et al. to investigate the effect of continuous intraperitoneal instillation of levobupivacaine in patients undergoing hysterectomy, the difference was not significant between the treatment and placebo groups on opioid consumption after surgery, length of stay in hospital, and pain score [28]. Also in RCT on 60 patients who underwent laparoscopic hysterectomy by Arden, mean pain, opioid consumption, and duration of hospitalization in the treatment (bupivacaine) and placebo (normal saline) groups were the same [29].

## 5. Conclusion

The use of intraperitoneal lidocaine combined with spinal anesthesia was not associated with a statistically significant difference in patients' postoperative VAS scores compared to spinal anesthesia and GA during and after gynecological surgical procedures.

## Figures and Tables

**Table 1:** Comparison of demographic characteristics and secondary outcomes between study groups.

	Group 1 (*n* = 28)	Group 2 (*n* = 28)	Group 3 (*n* = 28)	*P* value
Age (yr.)	29.62 ± (5.47)	29.51 ± (5.03)	29 ± (4.81)	0.88
Parity	0.47 ± (0.9)	0.48 ± (0.78)	0.37 ± (0.49)	0.83
Weight	72.9 ± (13.1)	69.9 ± (10.3)	75.8 ± (0.9)	0.76
Height	158.7 ± (5.2)	160.4 ± (5.9)	161.6 ± (2.5)	0.34
Analgesic usage				
Yes	12 (42.86)	10 (35.72)	11 (39.29)	0.82
No	16 (57.14)	18 (64.28)	17 (60.71)	
Vomiting				
Yes	8 (28.57)	7 (25)	7 (25)	0.94
No	20 (71.42)	21 (75)	21 (75)	
Duration of surgery	30.88 ± (11.25)	29.65 ± (10.6)	33.88 ± (19.13)	0.5
Indication for laparoscopy				
Infertility	22 (78.57)	21 (75)	23 (82.14)	0.87
Other indications for laparoscopy	6 (21.43)	7 (25)	5 (17.86)	

Values given as mean ± SD (standard deviation) or number (percentage) unless otherwise indicated; Group 1: general anesthesia, Group 2: spinal anesthesia, and Group 3: spinal anesthesia with lidocaine.

**Table 2:** The results of reported pain experienced at all-time interval.

Pain	Group 1 (*n* = 28)	Group 2 (*n* = 28)	Group 3 (*n* = 28)	*P* value^*∗*^
During surgery	—	2.75 ± (3.7)	2.25 ± (2.86)	0.57
After 2 hours	5.18 ± (3.66)	3.07 ± (3.4)	4.34 ± (3.58)	0.08
After 4 hours	4.69 ± (3.01)	3.38 ± (3.16)	4.15 ± (3.04)	0.27
After 6 hours	4.66 ± (3)	4.19 ± (3.13)	5.14 ± (3.02)	0.52
After 12 hours	4.36 ± (3.11)	4.96 ±(3.09)	3.96 ± (2.59)	0.47
Prior to discharge	3.3 ± (2.18)	3.65 ± (2.69)	2.62 ± (1.82)	0.24

^*∗*^The analysis of variance and independent *t*-test were used to compare mean between groups; Group 1: general anesthesia, Group 2: spinal anesthesia, and Group 3: spinal anesthesia with lidocaine.
